# Resistance of infection by *Plasmodium vivax* to chloroquine in Bolivia

**DOI:** 10.1186/s12936-015-0774-4

**Published:** 2015-07-01

**Authors:** Arletta Añez, Manuel Moscoso, Ángel Laguna, Cecilia Garnica, Viviana Melgar, Mauren Cuba, Sonia Gutierrez, Carlos Ascaso

**Affiliations:** Departamento de Salud Pública, Universidad de Barcelona, Barcelona, Spain; Organización Panamericana de Salud, Oficina de país Bolivia, La Paz, Bolivia; Laboratorio de control de calidad de medicamentos y toxicología del Instituto Nacional de laboratorios en Salud, CONCAMYT-INLASA, La Paz, Bolivia; Centro de Salud Rene Salazar, Gerencia de salud Riberalta-Beni, Beni, Bolivia; Centro de Salud La Unidad, Gerencia de salud Riberalta-Beni, Beni, Bolivia; Departamento de Salud Pública, Universidad de Barcelona, Institut d’Investicions Biomediques, Augusto Pi i Sunyer, Barcelona, Spain

**Keywords:** Chloroquine, *Plasmodium vivax*, Bolivia, Antimalarial drugs

## Abstract

**Background:**

Chloroquine (CQ) over three days plus primaquine (PQ) for seven days is the treatment of choice of infections by *Plasmodium vivax* in Bolivia, where 95% of the cases of malaria are attributed to this species. The aim of this study was to evaluate the therapeutic efficacy of CQ in this setting.

**Methods:**

Patients in the Amazon region of northern Bolivia, were included in the study from May to November 2011 and the therapeutic efficacy of CQ was evaluated over a 28-day follow-up period. Patients with *P. vivax* mono-infection received 25 mg/Kg body weight of CQ over three days. The concentrations of CQ + desethylchloroquine (DCQ) in blood were determined at days 7 and 28 of follow up; at follow-up and on the day of treatment failure was administered PQ.

**Results:**

One hundred patients fulfilled the inclusion criteria, two were lost to follow up and another two were later excluded for protocol violation. Of the 96 patients who completed the follow up 10 showed TF; one presented continued parasitaemia until day 7 of follow up, three on day 21 and six on day 28 of follow up. The geometric mean of CQ + DCQ on day 7 was 321.7 ng/ml (range 197–535 ng/ml). In six patients with TF the CQ + DCQ concentrations in blood on the day of TF were >100 ng/ml. The rate of resistance was 6.5%.

**Conclusion:**

The present study demonstrates the presence of resistance to CQ in the treatment of malaria by *P. vivax* in the Amazon region of Bolivia. New clinical trials are needed to establish alternative treatments against these parasites in this region of South America.

## Background

In South America, 60% [1] of the cases of malaria are due to *Plasmodium vivax* while, in Bolivia, 93% correspond to this parasite and the remaining 7% to *Plasmodium falciparum.* In Bolivia, the first-line therapeutic schedule for infections by *P. vivax* includes 25 mg/Kg body weight of chloroquine (CQ) for three days plus 3.5 mg of primaquine (PQ) for 7 days. The first is aimed at reducing the parasitic load in young forms of the parasite and eliminating immature gametocytes while the second drug is active against the gametocytes and hypnozoites of this species [2].

The first description of resistance to CQ in infections by *P. vivax* was in Papua New Guinea in 1989 [3], with the highest rates of resistance being reported on the same island of New Guinea [4, 5]. In Latin America, the first cases of resistance to CQ in infections by *P. vivax* were reported in Guyana in 1996, with three patients maintaining parasitaemia in the presence of adequate serum levels of CQ [6]. Since then cases of therapeutic failure (TF) have been described in different areas of the continent. However, confirmed resistance according to the serum levels of CQ and its main metabolite desethylchloroquine (DCQ) has only been reported in Peru with a rate of 1.2% [7] and in Brazil with 5.2% [8].

In 2003, an in vivo evaluation of resistance to CQ was carried out in the municipality of Riberalta in Bolivia, reporting 15% (9/59 cases) of TF over a 28-day period. However, none of the cases showed serum levels of the drug [9]. Another evaluation performed in 2007 in the same municipality showed TF of 6% (5/81), however CQ blood levels on the day of the evaluation were <100 ng/ml [10]. The present study is part of the routine monitoring of the therapeutic efficacy of anti-malarial drugs in Bolivia following the Pan-American Health Organization/World Health Organization (PHO/WHO) evaluation programme.

## Methods

### Study site

The study was developed in the department of Beni, in the municipality of Riberalta, in the north of the Amazonia of Bolivia where 18% of the diagnoses of malaria and 21% of the cases of malaria by *P. vivax* are reported in Bolivia [11].

### Study design

The screening methods for surveillance of anti-malarial drug efficacy (MSADE) of the WHO [12] were followed. The sample size was defined as established in MSADE determined by the proportion of TF in South America and corresponding to no more than 5%, with a confidence interval of 95% and a precision of 5%. Thus, 73 patients were to be included with a maximum period of seven months. The patients were recruited and followed by multidisciplinary teams in two health care centers: La Unidad health care centre (LU) and the Cesar Moscoso health care centre (CM) in the urban area of the Riberalta, being equal distance from each other (3,000 m) The inclusion criteria were: patients greater than five years of age diagnosed with mono-infection with *P. vivax*, a parasite density of asexual forms of 250–100,000 parasites/μl of blood, a temperature >37.5°C or in its absence a history of a rise in temperature within the last 48 h. Microscopic diagnosis was performed according to the practical guidelines of diagnosis of the National Malaria Control Programme [13], (thick blood film, Giemsa staining) and parasite density was calculated per microlitre of blood by dividing the number of parasites counted multiplied by 6,000, divided by leucocytes counted. The results were obtained from the average of two experienced microscopists. In the case of a difference in parasitic density greater than 50%, the final result was considered by averaging the result of a third microscopist. The presence of gametocytes was also determined in this way.

The exclusion criteria were: pregnant women confirmed by rapid pregnancy diagnostic tests (detection of human chorionic gonadotropin in urine), patients with signs and symptoms of severe malaria, a history of allergy to anti-malarial drugs, concomitant presence of severe or chronic diseases such as tuberculosis or HIV/AIDS and a previous history of having received anti-malarial drugs.

The treatment consisted of the use of chloroquine phosphate (Lote FCV 002A, Macleods Pharmaceuticals Ltd, India) 25 mg/Kg body weight administered orally over 3 days: on days 0 and 1 of the study 10 mg/Kg body weight were given and on day 2, 5 mg/Kg body weight were administered under strict supervision of the investigative team. Patients vomiting within half an hour after the administration of the drug were given another treatment cycle with the same dose and patients with more than two episodes of vomiting were excluded from the study.

The patients who accepted to participate in the study were followed on days 2, 3, 7, 14, 21 and 28 after the treatment. The day of diagnosis was denominated day 0. After the 28 days of follow-up all the patients were treated with PQ 0.5 mg/Kg body weight/day administered orally during seven days. Patients with TF or those in whom mixed malaria was detected were excluded from the study. These patients were then administered artemisinin-based combination therapy (ACT) used in Bolivia for the treatment of *P. falciparum*, plus PQ 0.5 mg/Kg over 7 days.

Two milliliters of blood were obtained by venopuncture from all the patients in the study, with or without parasitaemia, on days 7 and 28 of the follow up. The concentrations of CQ and DCQ in blood were also determined by high performance liquid chromatography (HPLC) according to the standardized technique of the Center for Disease Control in Atlanta, USA in patients with TF. The samples obtained were kept at 4°C until analysis.

The HPLC was performed using the Shimadzu LC-10ATVP with RF-10 AXL fluorescence detector under the following chromatographic conditions: chromatographic column: Agilent Zorbax SIL, silane packing (corresponding to L3 according to USP). Detector: fluorescence, emission 380 nm, excitation 320 nm, mobile phase A: methanol and diethylamine (100:0.3); mobile phase B: *n*-hexane, *t*-butyl methyl ether and diethylamine (1:1:0.003), flow gradient: 1.0 ml/min; injection volume 7 µl and column temperature: 30°C. A concentration of CQ + DCQ of 70–99 ng/ml was considered effective to eliminate all the asexual and sexual forms of *P. vivax* in blood [14] and a CQ + DCQ concentration >100 ng/ml in the presence of parasitaemia was considered to demonstrate resistance to CQ [15–17].

### Statistical analysis

The Tableau^®^ 7 (Professional edition) software was used to analyse the data of frequency and to measure the data of CQ + DCQ. The statistical package SPSS^®^ v 20 (Chicago, IL, USA) 14 was used to compare the means and medians of the results of CQ + DCQ and associated variables, with parametric and non-parametric models of analysis of variance. Kaplan-Meier analysis of survival and confidence intervals was analysed by the WHO programme for study the *in vivo* therapeutic efficacy [12]. All the contrasts of hypothesis were evaluated with an alpha risk of 5% and the estimations were made with a confidence interval of 95%.

### Ethical aspects

The study was performed following the recommendations of the National Committee of Bioethics of Bolivia and the Ethical Committee of PAHO (PAHOERC). All patients provided informed consent to participate in the study, and in those under legal age informed consent was obtained from their legal guardians.

## Results

From May to November 2011, 656 cases of malaria by *P. vivax* were reported out of a total of 5,290 cases evaluated in the urban area of Riberalta. One hundred of these patients (51 in CM and 49 in LU) were included in the study. Of the total number of cases evaluated 64% were males and 36% females, with a median age of 20 years (range 5–69 years), and 25% of whom were under the age of 15 years. Thirty-one percent (n = 31) had an axillary temperature >37°C, the geometric mean of PD on day 0 was 3,837.43 parasites/µl (range 252.35–29,987.15 parasites/µl), The geometric mean of CQ administered over the three days of treatment was 1,332.19 mg (range 375–2,250 mg). The mean haemoglobin values on day 0 in the females was 10 g/dl (range 7–14 g/dl) and 12 g/dl (range 8–16 g/dl) in males, and 75% (n = 75) had anaemia before initiating the treatment (Table 1).

**Table 1:** Characteristics of the patients included in the study

Characteristics	
Total number of patients recruited	100
Age	
Median age (year, range)	20 (5–69)
Number of patients <15 years of age	25% (n = 25)
Sex	
Female	36% (n = 36)
Male	64% (n = 64)
History of fever	97% (n = 97)
Axillary temperature >37°C (day 0)	31% (n = 31)
Geometric mean of parasites/μl (day 0) (CI 95%)	3,837.43 (252.35–29,987.15)
Geometric mean CQ concentration in mg at day 3 (CI 95%)	1,332.19 (375-2,250)
Mean haemoglobin value (day 0)	
Female, g/dl; range	10 (7–14)
Male, g/dl; range	12 (8–16)
No. (%) with anaemia^a^	75 (75%)

^a^Haemoglobin level <12 g/dl for females and <13 g/dl for males (according to WHO criteria).

### Response to treatment

Of the 100 patients included in the study two were lost to follow up and another two were later excluded; one for presenting co-infection by *P. falciparum* and the other for violating the protocol. Therefore, a total of 96 patients were followed until day 28 after treatment. Of these 96 patients 59% (n = 57) eliminated the parasitaemia on day 2; 91% (n = 87) on day 3 and 99% (n = 95) on day 7 of follow up. Parasitaemia or clinical deterioration were not observed until the end of follow up in 89.6% (n = 86). Treatment failure was observed on days 7, 21 and 28 follow-up, with 1.04% (n = 1) on day 7, 3.1% (n = 3) on day 21 and 6.3% (n = 6) on day 28. The median age of the patients cured was 21 years (range 5–61 years) and that of patients with TF was 14 years (range 5–32 years), with the differences being statistically significant (P = <0.05). With the Kaplan-Meier analysis the cumulative incidence of treatment failure was 0.104 (95% CI 0.057–0.185).

### Blood concentrations of CQ + DCQ

**Figure 1:**
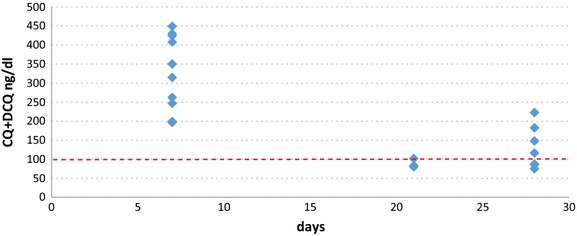
Chloroquine + desethylchloroquine (CQ + DCQ) concentrations in blood on day 7 (D7). Follow and on the day of therapeutic failure (DTF) in the 10 patients with therapeutic failure.

The geometric mean CQ + DCQ value in blood of all the patients followed with and without TF (n = 96) on day 7 was 319.46 ng/ml (range 50–743 ng/ml), being 109.24 ng/ml (range 34–602 ng/ml) on day 28 of follow up (n = 92). In the patient with continued presence of parasites in blood until day 7 inclusive the CQ + DCQ values on day 7 were 535 µg/ml and the patient was excluded from the study. In the 10 patients presenting TF the geometric mean of CQ + DCQ was 321.7 ng/ml (range 197–535 ng/ml) on day 7, being 113.46 ng/ml (range 75–223 ng/ml) on the day of TF. In six patients the CQ + DCQ level on the day of TF was above the minimum elimination concentration (MEC) (100 ng/ml) (Table 2; Figure 1). Using Kaplan-Meier survival analysis, the cumulative incidence of therapeutic failure due to resistance was 0.65 (95% CI 0.029–0.139) (Table 3).

**Table 2:** Results on the day of therapeutic failure (TF)

Center	Day TF	Age years	Weight (Kg)	CQ administered mg	CQ + DCQ D7^a^	CQ + DCQ TF^b^
LU 10	7	32	62	1.500	535	
CM 02	21	17	72	1.875	351	102
CM 35	21	9	29	750	247	80
LU 07	21	7	21	525	449	83
CM 42	28	5	16	450	263	117
CM 23	28	14	56	1.500	315	183
CM 43	28	19	61	1.575	197	148
LU 12	28	14	57	1.500	431	223
LU 28	28	14	42	1.050	408	86
LU 38	28	9	27	675	199	75

Concentrations of CQ + DCQ on day 7 and on the day of TF.

^a^CQ + DCQ D7 chloroquine + desethylchloroquine on day 7 of follow up.

^b^CQ + DCQ TF chloroquine + desethylchloroquine on the day of therapeutic failure.

**Table 3:** Estimation of cumulative incidence (risk) of current parasitaemia after chloroquine therapy and malaria by *P. vivax* adjusted for the concentration of chloroquine + desethylchloroquine (CQ + DCQ) in blood on the day of therapeutic failure (TF)

Day	Number of patients	TF/CQ + DCQ >100 ng/dl	Censored	Failure cumulative incidence
0	96	0	0	0
2	96	0	3	0
3	93	0	0	0
7	93	1	1	0.01
14	91	0	0	0.01
21	91	1	0	0.022
28	90	4	0	0.065
Total	86	6	4	(95% CI 0.029–0.139)

## Discussion

According to the MSADE [12], the presence of parasites from day 7 to 28 of follow up at CQ + DCQ blood concentrations >100 ng/ml indicates resistance to CQ. Thus, in the Amazon region of Bolivia there is evidence of resistance to CQ in *P. vivax* infections.

The concentration of CQ-DCQ in blood on day 7 in all the patients with TF was above the MEC of the drug. In addition, the geometric mean was 321.7 ng/ml (range 197–535 ng/ml) demonstrating the CQ presented adequate absorption through the intestinal tract [18]. In 6 of the 10 patients with TF it was observed that the CQ + DCQ levels on the day of TF were >100 ng/ml using Kaplan-Meier survival analysis, the cumulative incidence of therapeutic failure due to resistance was 0.65 (95% CI 0.029–0.139).

Ruebsh et al. confirmed resistance to CQ in infections by *P. vivax* with the levels of the drug in blood, describing resistance in two out of 177 patients in the Amazon region of Peru [7]. Santana Filho reported parasitaemia above the MEC of the drug in 11 out of 109 patients in the Amazon region of Brazil, although DCQ concentrations were not determined [19]. In addition, Marquez et al. described resistance in seven out of 135 patients receiving CQ + PQ in the same Amazon region of Brazil [8].

According to the recommendations of the WHO, a change in the treatment schedule should be considered in cases of proven resistance >10% [2]. The rate of resistance to CQ in Latin America is rising [20] and treatment alternatives should be investigated to achieve better control against malaria in this region. At present combined treatments with ACT are recommended for infections by *P. falciparum* because of their demonstrated rapid effectiveness in eliminating the parasitic load and achieving complete cure of the disease [2]. This is the alternative treatment which should be implemented in the treatment of malaria by *P. vivax.* Several clinical trials have demonstrated the efficacy of the ACT in the treatment of malaria *P. vivax*, with dihydroartemisinin-piperaquine being the ACT most frequently studied [21]. Taking into account the low prevalence of glucose 6 phosphate dehydrogenase deficiency in the Amazon region of South America [22], the chosen ACT could be combined with PQ [23, 24], with the aim of preventing a recurrence of parasitaemia due to circulating hypnozoites, however further studies are still required to use these drugs simultaneous.

## Conclusions

In conclusion, 6.5% of resistance to CQ was observed in infections by *P. vivax* in the Amazon region of Bolivia. Considering the percentage of resistance found and the rate of TF with MEC of the drug in blood on the day of TF this resistance could be greater. New treatment schedules should be evaluated to guarantee the control of malaria in this region.

## Abbreviations

CQ: chloroquine
DCQ: desethylchloroquine
PQ: primaquine
TF: treatment failure
ACT: artemisinin-based combination therapy
MEC: minimum effective concentration
LU: La Unidad health care centre
CM: Cesar Moscoso health care centre
PD: parasitic density
MSADE: screening methods for surveillance of anti-malarial drug efficacy
